# Mitral annular disjunction in out-of-hospital cardiac arrest patients—a retrospective cardiac MRI study

**DOI:** 10.1007/s00392-024-02440-3

**Published:** 2024-04-11

**Authors:** Felix Troger, Gert Klug, Paulina Poskaite, Christina Tiller, Ivan Lechner, Martin Reindl, Magdalena Holzknecht, Priscilla Fink, Eva-Maria Brunnauer, Elke R. Gizewski, Bernhard Metzler, Sebastian Reinstadler, Agnes Mayr

**Affiliations:** 1 University Clinic of Radiology, Medical University of Innsbruck, Anichstrasse 35, 6020 Innsbruck, Austria; 2 University Clinic of Internal Medicine III, Cardiology and Angiology, Medical University of Innsbruck, Anichstrasse 35, 6020 Innsbruck, Austria

**Keywords:** Mitral valve, Mitral annular disjunction, Out-of-hospital cardiac arrest, Cardiac magnetic resonance imaging

## Abstract

**Background:**

Mitral annular disjunction (MAD), defined as defective attachment of the mitral annulus to the ventricular myocardium, has recently been linked to malignant arrhythmias. However, its role and prognostic significance in patients requiring cardiopulmonary resuscitation (CPR) remain unknown. This retrospective analysis aimed to describe the prevalence and significance of MAD by cardiac magnetic resonance (CMR) imaging in out-of-hospital cardiac arrest (OHCA) patients.

**Methods:**

Eighty-six patients with OHCA and a CMR scan 5 days after CPR (interquartile range (IQR): 49 days before – 9 days after) were included. MAD was defined as disjunction-extent ≥ 1 mm in CMR long-axis cine-images. Medical records were screened for laboratory parameters, comorbidities, and a history of arrhythmia.

**Results:**

In 34 patients (40%), no underlying cause for OHCA was found during hospitalization despite profound diagnostics. Unknown-cause OHCA patients showed a higher prevalence of MAD compared to definite-cause patients (56% vs. 10%, *p* < 0.001) and had a MAD-extent of 6.3 mm (IQR: 4.4–10.3); moreover, these patients were significantly younger (43 years vs. 61 years, *p* < 0.001), more often female (74% vs. 21%, *p* < 0.001) and had fewer comorbidities (hypertension, hypercholesterolemia, coronary artery disease, all *p* < 0.005). By logistic regression analysis, the presence of MAD remained significantly associated with OHCA of unknown cause (odds ratio: 8.49, 95% confidence interval: 2.37–30.41, *p* = 0.001) after adjustment for age, presence of hypertension, and hypercholesterolemia.

**Conclusions:**

MAD is rather common in OHCA patients without definitive aetiology undergoing CMR. The presence of MAD was independently associated to OHCA without an identifiable trigger. Further research is needed to understand the exact role of MAD in OHCA patients.

**Graphical Abstract:**

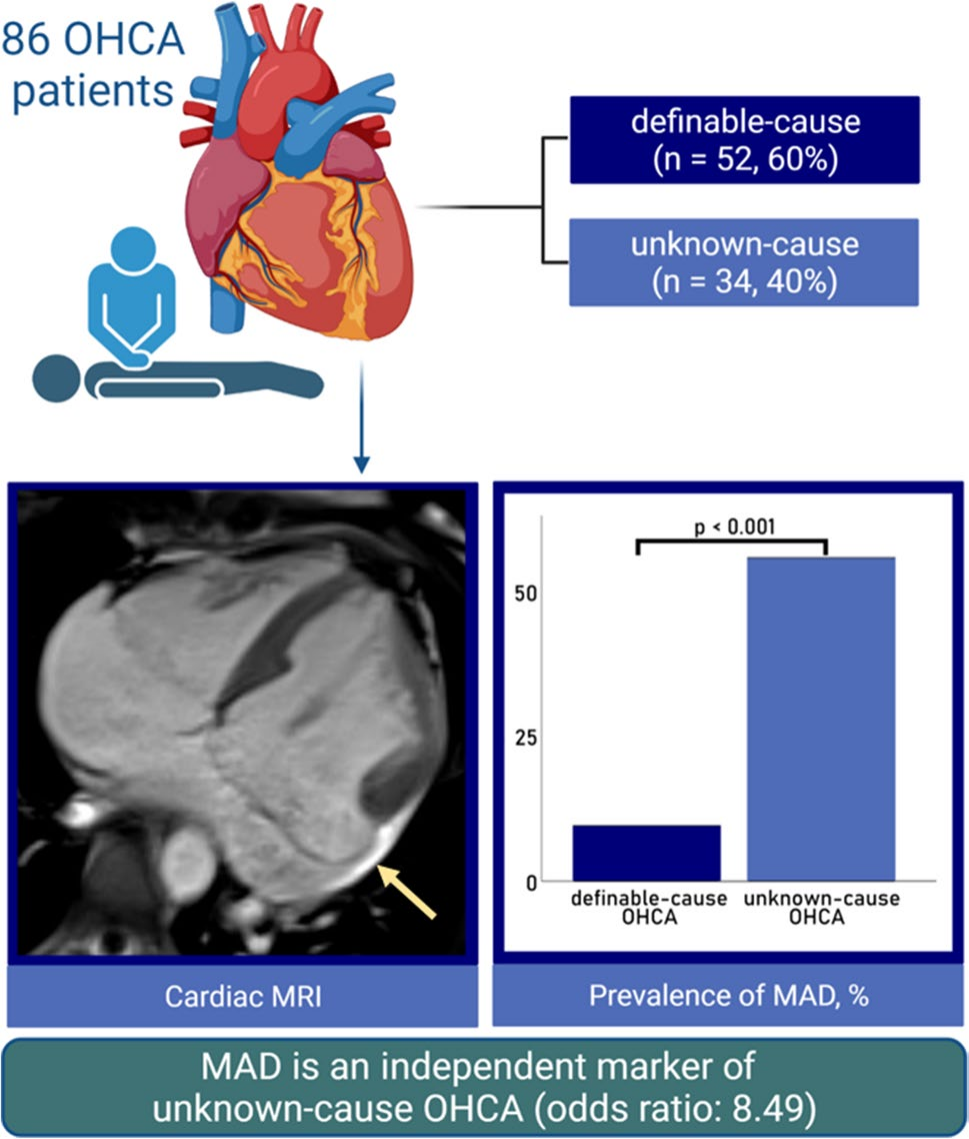

Study synopsis - MAD occurs frequently in unknown-cause OHCA and represents an independent marker after adjustment for age, hypertension, and hypercholesterolemia. (Illustration created with biorender.com). MAD mitral annular disjunction, OHCA out-of-hospital cardiac arrest

**Supplementary Information:**

The online version contains supplementary material available at 10.1007/s00392-024-02440-3.

## Introduction

Mitral annular disjunction (MAD) represents the defective anchoring of the mitral valve annulus into the ventricular myocardium [1]. This anatomical variant has long been regarded as rather common but clinically irrelevant secondary finding to mitral valve prolapse (MVP) [2]. However, its status as distinct disease entity, acting as possible substrate for ventricular arrhythmias, has been increasingly substantiated within the past few years [3, 4]. In 2019, a case report by Bennett et al. was the first to describe MAD as the only true contributing factor to cardiac arrest in a 38-year-old otherwise healthy patient [5]. Recent studies suggested that its formerly assumed prevalence has been clearly underestimated [6]; additionally, the term MAD was uncoupled from its status as a negligible auxiliary finding of MVP, as it was shown that MAD could be detected even without concomitant prolapse [3]. To date, research interest in MAD is continuously growing [2]. However, data about its clinical relevance and postulated association to ventricular arrhythmias are scarce [5, 7, 8]. Furthermore, data about prevalence and significance in out-of-hospital cardiac arrest (OHCA) patients are completely lacking. Nevertheless, OHCA represents a leading cause of mortality worldwide [9], with an estimated 20% being of unknown or unobtainable cause [10]. Although most studies tend to use echocardiography to screen and evaluate MAD, assessment by cardiac magnetic resonance (CMR) imaging seems more appropriate in MAD screening due to its higher sensitivity, especially in MAD of minor extent [11].

Accordingly, the aims of this retrospective study were as follows: (a) to determine the prevalence of MAD in a population of OHCA patients, (b) to assess its prevalence in OHCA patients in whom no definite cause of cardiac arrest (CA) was finally definable, and (c) to classify the role of MAD in this latter patient group.

## Methods

### Study population

**Fig. 1:**
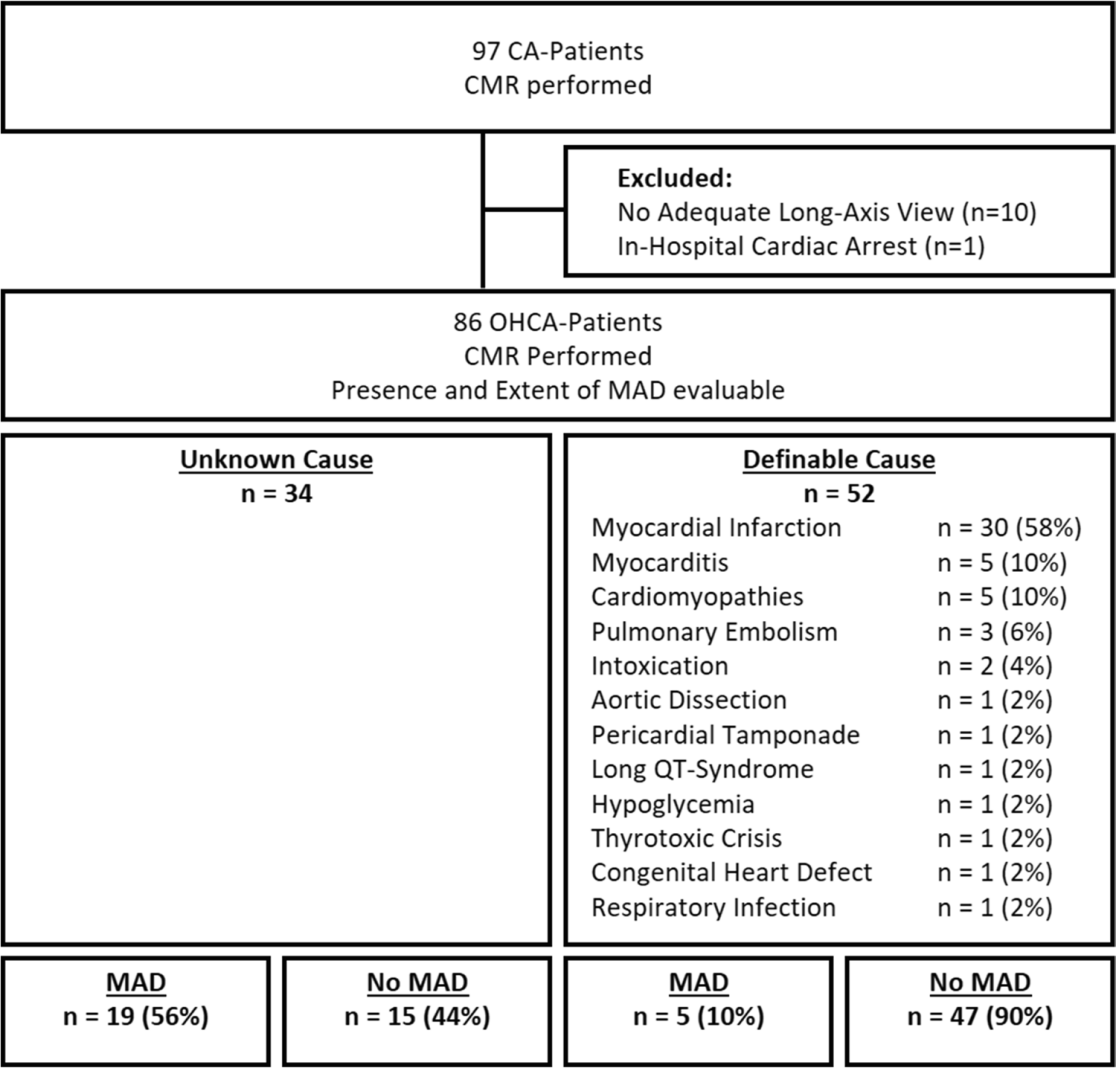
Flowchart of in- and exclusion, displaying the definable causes of CPR. CA cardiac arrest, CMR cardiac magnetic resonance imaging, CPR cardiopulmonary resuscitation, MAD mitral annular disjunction, OHCA out-of-hospital cardiac arrest

The study population included all OHCA patients treated at the local university hospital from June 2007 to April 2021, where an adequate CMR scan was available. Patient records were screened for comorbidities and risk factors, positive family history for coronary artery disease (CAD) or CA and laboratory parameters as well as further diagnostics, including electrocardiography (ECG) and cardiac computed tomography (CT). Moreover, these records were checked for additional rhythmological events before, during, or after hospital stay. CAD was defined as any coronary atherosclerotic disease detected in the respective modality (i.e. CT or cardiac catheterization). A flowchart of in- and excluded patients is shown in Fig. 1. This study was approved by the local Ethics Committee and conforms to the Declaration of Helsinki.

### Cardiovascular magnetic resonance imaging

All CMR scans were performed on a 1.5T clinical MR-scanner (MAGNETOM Avanto or Avantofit; Siemens Healthineers AG, Erlangen, Germany). The standard CMR protocol used in this study can be found in the supplement. To calculate body surface area, the Du Bois formula was used [12].

MAD was defined as the presence of detachment ≥ 1 mm between the mitral annulus and ventricular myocardium, affecting the area under the posterior valve leaflet [3]. The extent of MAD was measured longitudinally as the distance from atrial valve leaflet junction to the top of the LV myocardium at end-systole in long-axis cine-images. Only patients with a CMR of sufficient quality to decide whether MAD is present or not were included in this study, in order to avoid false-positive diagnoses of MAD (so-called ‘pseudo-MAD’ feigned by juxtaposition of the posterior leaflet [13]). To determine the particular affected mitral segments, suitable short-axis slices were used.

**Fig. 2:**
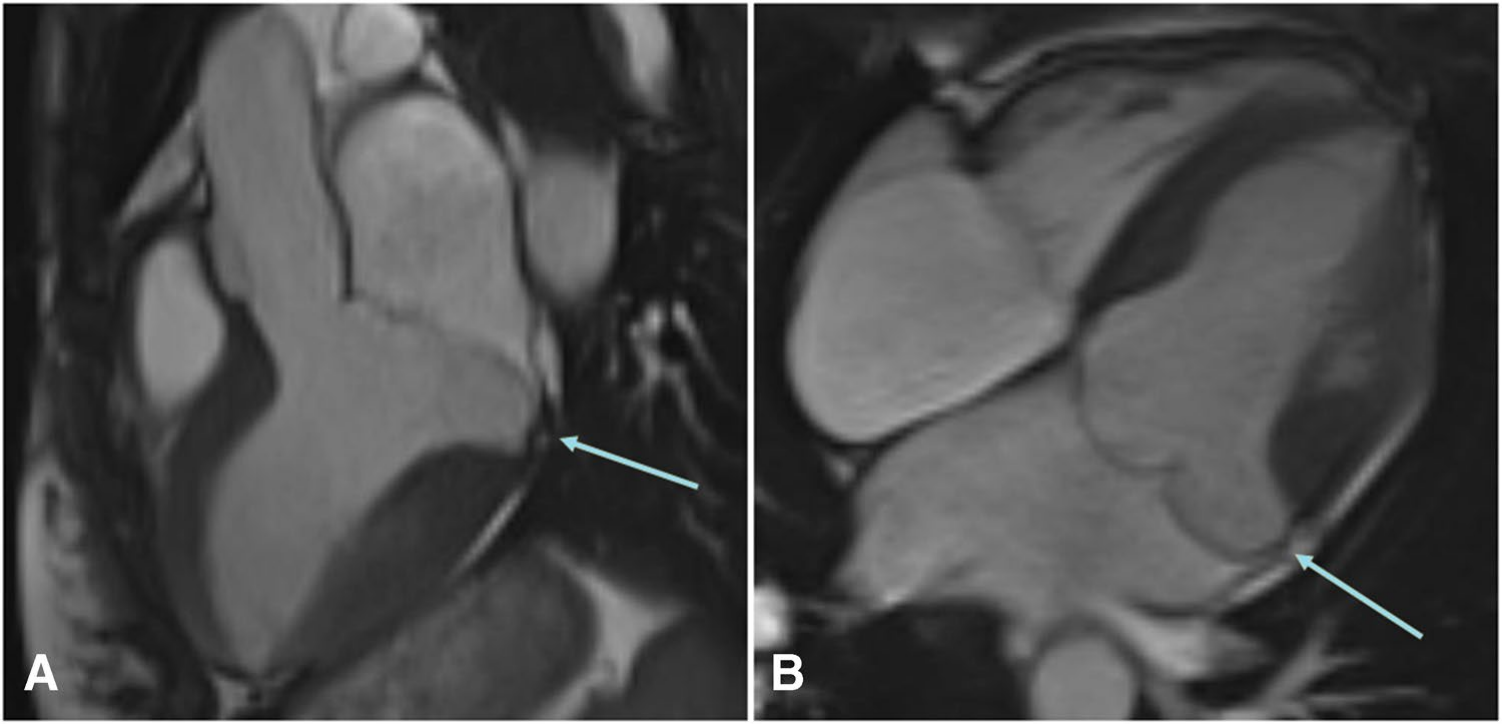
Three- (**A**) and four-chamber (**B**) view of a 27-year-old female patient with out-of-hospital cardiac arrest of unknown cause, showing a distinct mitral annular disjunction with systolic curling (arrow) of the P2- and P1-segment of the mitral valve with a maximum extent of 14.2 mm

MVP was defined as superior displacement ≥ 2 mm of any part of the mitral leaflet beyond the mitral annulus [3, 14]. Systolic curling motion was defined as unusual systolic motion of the posterior mitral ring on the adjacent myocardium [15], as illustrated in Fig. 2. MAD-presence and -extent as well as presence of MVP were conducted in full by two independent observers blinded to clinical data, each with several years of experience in CMR diagnosis (AM, 13 years, EuroCMR-level II-certified; FT, 3 years).

### Statistical analysis

SPSS Statistics 26.0 (IBM, Armonk, NY, USA) was used for statistical analyses. Results for continuous variables are expressed as medians with corresponding IQR, categorical variables as absolute numbers and percentages. Differences in continuous and categorical variables between the two groups were tested by the Mann–Whitney U test and the chi-square test, respectively. A* p*-value < 0.05 was considered as statistically significant. Logistic regression analysis was performed to evaluate independent markers for OHCA of unknown cause as well as independent markers for MAD; variables with a *p*-value < 0.10 in univariable analysis and within these the variables of clinical relevance were included in our multivariable models.

## Results

### Baseline patient characteristics

For the current retrospective analysis, 86 OHCA patients were included, with a regain of spontaneous circulation after 15 min (IQR: 5–25, available in 81 patients) and a shockable rhythm in 72 patients (of 81 acute ECG data available). All patients underwent cardiopulmonary resuscitation (CPR) at a median age of 56 years (IQR: 41–67). CMR was performed 5 days after resuscitation (IQR: 49 days before – 9 days after). At discharge, no definite reason for CA was found after excluding coronary/cardiac, infectious, thromboembolic, genetic/congenital, or metabolic conditions as well as intoxications, in 34 patients (40%). These patients are referred to as ‘unknown-cause OHCA’. Patient inclusion criteria as well as the particular causes for CA are listed in Fig. 1. Baseline characteristics are shown in Table 1.

**Table 1:** Baseline characteristics

	All patients (*n* = 86)	Definable-cause (*n* = 52)	Unknown-cause (*n* = 34)	*p*-value
Age at CPR, years	56 (41–67)	61 (54–69)	43 (33–55)	** < 0.001**
Female, %	36 (42)	11 (21)	25 (74)	** < 0.001**
BMI, kg/m^2^	25 (22–28)	26 (23–29)	24 (21–27)	**0.007**
MAD, *n* (%)	24 (28)	5 (10)	19 (56)	** < 0.001**
MAD – no. of affected segments				
- 0	62 (72)	47 (90)	15 (44)	** < 0.001**
- 1	13 (15)	4 (8)	9 (26)	0.098
- ≥ 2	11 (13)	1 (2)	10 (29)	**0.001**
MAD-extent, mm (24/86 patients)	5.7 (4.5–10.3)	5.4 (3.8–9.0)	6.3 (4.4–10.3)	0.534
Systolic curling, *n* (%) (24/86 patients)	8/24 (33)	1/5 (20)	7/19 (37)	0.477
MVP, *n* (%)	26 (30)	13 (25)	13 (38)	0.191
- Anterior	14 (16)	9 (17)	5 (15)	0.993
- Posterior	3 (3)	2 (3)	1 (3)	0.998
- Bileaflet	9 (10)	2 (3)	7 (21)	0.100
Risk profile				
Smoker, *n* (%)	27 (31)	18 (35)	9 (26)	0.442
Diabetes, *n* (%)	11 (13)	9 (17)	2 (6)	0.090
Hypertension, *n* (%)	41 (48)	32 (62)	9 (26)	**0.001**
Hypercholesterolemia, *n* (%)	29 (34)	24 (46)	5 (15)	**0.003**
Positive family history, *n* (%)	10 (12)	6 (12)	4 (12)	0.961
Atrial fibrillation, *n* (%)	7 (8)	7 (13)	0 (0)	**0.029**
CAD (CT or invasively, 83/86 patients), *n* (%)	44/83 (53)	37/51 (73)	7/32 (24)	** < 0.001**
Laboratory parameters				
Admission glucose, mg/dl	167 (125–235)	162 (125–246)	173 (121–218)	0.848
Creatinine, mg/dl	1.03 (0.85–1.24)	1.15 (0.93–1.43)	0.90 (0.76–1.05)	** < 0.001**
Sodium, mmol/l	139 (138–141)	139 (137–141)	140 (138–142)	0.558
Potassium, mmol/l	3.8 (3.5–4.3)	4.0 (3.5–4.3)	3.7 (3.5–4.2)	0.256
Calcium, mmol/l	2.2 (2.1–2.3)	2.2 (2.1–2.3)	2.1 (2.0–2.2)	**0.009**
Hemoglobin, g/l	136 (126–149)	139 (128–151)	132 (121–144)	0.315
Leukocytes, G/l	11 (8–14)	11 (8–15)	11 (8–14)	0.931
CRP, mg/dl	0.3 (0.1–1.2)	0.3 (0.1–1.7)	0.2 (0.1–0.7)	0.329
INR	1.1 (1.0–1.2)	1.1 (1.0–1.2)	1.1 (1.1–1.3)	0.363
Admission hs Troponin T, ng/ml	43 (17–191)	53 (20–306)	41 (15–181)	0.327
Peak hs-Troponin T, ng/ml	223 (119–1334)	598 (149–3874)	189 (104–441)	**0.017**
Admission CK, U/l	156 (87–341)	153 (79–343)	156 (96–345)	0.686
Peak CK, U/l	740 (167–2032)	768 (137–2645)	727 (273–1163)	0.927
Peak NT-proBNP, ng/l	710 (311–1805)	1050 (394–3050)	485 (182–1326)	**0.048**
CMR parameters				
LV EF, %	47 (38–53)	41 (33–50)	49 (43–56)	**0.030**
LV EDVi, ml/m^2^	100 (80–117)	106 (88–132)	91 (73–110)	**0.027**
LV ESVi, ml/m^2^	52 (41–67)	58 (49–85)	48 (34–59)	**0.015**
LV myocardial mass, g	116 (97–147)	142 (111–168)	110 (85–121)	** < 0.001**
RV EF, %	50 (39–56)	45 (34–54)	52 (46–57)	0.096
RV EDVi, ml/m^2^	87 (71–106)	94 (73–109)	81 (68–103)	0.203
RV ESVi, ml/m^2^	45 (33–56)	49 (42–58)	43 (31–50)	0.064
LGE (82/86 patients), *n* (%)	39/82 (48)	36/50 (72)	3/32 (9)	** < 0.001**

*BMI* body mass index, *CAD* coronary artery disease, *CK* creatine kinase, *CPR* cardiopulmonary resuscitation, *CRP* C-reactive protein, *CT* computed tomography, *EDVi* indexed end-diastolic volume, *EF* ejection fraction, *ESVi* indexed end-systolic volume, *hs* high-sensitive, *INR* international normalized ratio, *LGE* late gadolinium enhancement, *LV* left ventricular, *MAD* mitral annular disjunction, *MVP* mitral valve prolapse, *NT-proBNP* N-terminal pro-B-type natriuretic peptide, *RV* right ventricular

*p*-values of statistical significance are printed in bold

### Mitral annular disjunction

Overall, MAD was present in 28% of OHCA patients (*n* = 24), with a median MAD-extent of 5.7 mm (IQR: 4.5–10.3), ranging from 2.8 to 14.3 mm. Patients with MAD were significantly younger (40 years [IQR: 32–52] vs. 61 years [IQR: 50–70], *p* < 0.001) and more often female (75% vs. 29%, *p* < 0.001). Moreover, MAD patients had lower body mass index (BMI; 23kg/m^2^ [IQR: 20–26] vs. 26kg/m^2^ [IQR: 23–29], *p* = 0.006) and a lower prevalence of diabetes, hypertension, and hypercholesterolemia (all *p* < 0.03). Within MAD patients, 8 showed systolic curling motion (33%). MVP was present in 26 patients (30%; with MAD: *n* = 15 [63%] vs. without MAD: *n* = 11 [18%], *p* < 0.001) with a median extent of 4 mm (IQR: 3–6). A detailed comparison of patients with and without MAD is shown in Table 2.

**Table 2:** Comparison of patients with and without MAD

	MAD (*n* = 24)	No MAD (*n* = 62)	*p*-value
Age at CPR, years	40 (32–52)	61 (50–70)	** < 0.001**
Female, %	18 (75)	18 (29)	** < 0.001**
BMI, kg/m^2^	23 (20–26)	26 (23–29)	**0.006**
MVP, *n* (%)	15 (63)	11 (18)	** < 0.001**
- Anterior	7 (29)	7 (11)	0.261
- Posterior	1 (4)	2 (3)	0.998
- Bileaflet	7 (29)	2 (3)	**0.007**
Risk profile			
Smoker, *n* (%)	6 (25)	21 (34)	0.375
Diabetes, *n* (%)	0 (0)	11 (18)	**0.024**
Hypertension, *n* (%)	5 (21)	36 (58)	**0.001**
Hypercholesterolemia	3 (13)	26 (42)	**0.007**
Positive family history, *n* (%)	2 (8)	8 (13)	0.523
Atrial fibrillation, *n* (%)	0 (0)	7 (11)	0.078
CAD (CT or invasively, 83/86 patients), *n* (%)	4/23 (17)	40/60 (67)	** < 0.001**
Laboratory parameters			
Admission glucose, mg/dl	166 (120–202)	167 (125–246)	0.368
Creatinine, mg/dl	0.84 (0.72–1.01)	1.13 (0.92–1.41)	** < 0.001**
Sodium, mmol/l	140 (138–141)	139 (137–142)	0.315
Potassium, mmol/l	3.7 (3.5–4.0)	3.9 (3.5–4.3)	0.240
Calcium, mmol/l	2.1 (2.0–2.2)	2.2 (2.1–2.3)	**0.021**
Hemoglobin, g/l	131 (117–139)	140 (128–151)	**0.032**
Leukocytes, G/l	12 (7–15)	11 (8–14)	0836
CRP, mg/dl	0.1 (0.1–1.2)	0.3 (0.1–1.2)	0.238
INR	1.1 (1.0–1.3)	1.1 (1.0–1.2)	0.829
Admission hs Troponin T, ng/ml	70 (14–187)	37 (19–216)	0.777
Peak hs-Troponin T, ng/ml	200 (126–681)	267 (104–3175)	0.399
Admission CK, U/l	187 (103–521)	151 (79–333)	0.164
Peak CK, U/l	921 (361–2067)	504 (143–2032)	0.182
Peak NT-proBNP, ng/l	402 (131–907)	1064 (431–3034)	**0.003**
CMR parameters			
LV EF, %	47 (43–56)	45 (33–52)	0.111
LV EDVi, ml/m^2^	94 (80–111)	103 (79–126)	0.376
LV ESVi, ml/m^2^	49 (40–58)	56 (41–77)	0.158
LV myocardial mass, g	102 (85–118)	125 (110–160)	**0.006**
RV EF, %	54 (46–58)	48 (34–54)	**0.043**
RV EDVi, ml/m^2^	85 (71–104)	89 (71–109)	0.685
RV ESVi, ml/m^2^	43 (32–49)	46 (33–58)	0.129
LGE (82/86 patients), *n* (%)	5/24 (21)	34/58 (59)	**0.002**

*BMI* body mass index, *CAD* coronary artery disease, *CK* creatine kinase, *CPR* cardiopulmonary resuscitation, *CRP* C-reactive protein, *CT* computed tomography, *EDVi* indexed end-diastolic volume, *EF* ejection fraction, *ESVi* indexed end-systolic volume, *hs* high-sensitive, *INR* international normalized ratio, *LGE* late gadolinium enhancement, *LV* left ventricular, *MAD* mitral annular disjunction, *MVP* mitral valve prolapse, *NT-proBNP* N-terminal pro-B-type natriuretic peptide, *RV* right ventricular

*p*-values of statistical significance are printed in bold

### Unknown-cause out-of-hospital cardiac arrest

Patients without definite substrate for CA were significantly younger (43 years [IQR: 33–55] vs. 61 years [IQR: 54–69], *p* < 0.001) and more often female (*n* = 25 [74%] vs. *n* = 11 [21%], *p* < 0.001). Nineteen OHCA patients without definite cause for CA had MAD (56%) with a median MAD-extent of 6.3 mm (IQR: 4.4–10.3); of these, 10 patients (53%) with MAD had two or three mitral segments affected. Unknown-cause OHCA patients had lower BMI (24 kg/m^2^ [IQR: 21–27] vs. 26 kg/m^2^ [IQR: 23–29], *p* = 0.007]) and a lower prevalence of arterial hypertension (26% vs. 62%, *p* = 0.001) and hypercholesterolemia (15% vs. 46%, *p* = 0.003). Before hospitalization, atrial fibrillation occurred only in patients with a definite cause for CA (*n* = 7, 13%).

A total of 83 OHCA patients (97%) were evaluated for the presence of CAD, either by coronary angiography (performed in 63 patients (76%) on the day of CPR [IQR: 0–6 days after]) or by coronary computed tomography angiography (CTA) (in 52 patients (60%) performed on the day of CPR [IQR: 2 days before – 1 day after]). Combined, these two modalities resulted in an overall CAD prevalence of 53%. CAD was significantly more common in patients with a definable cause for OHCA (73% vs. 24%, *p* < 0.001).

MAD was shown to be significantly associated with unknown-cause OHCA univariably (odds ratio (OR): 11.91, 95% confidence interval (CI): 3.79–37.37, *p* < 0.001) and to be an independent marker of unknown-cause OHCA after adjustment for age, hypertension, and hypercholesterolemia (OR: 8.49, 95% CI: 2.37–30.41, *p* = 0.001) by logistic regression analysis. Results of uni- and multivariable analyses are listed in supplementary Table 1.

### CMR measurements

In unknown-cause OHCA patients (*n* = 34, 40%), CMR was performed 6 days after CPR (IQR: 6–8). In 6 of these, CMR was performed before CPR, with specific indications including evaluation of ventricular extrasystoles (*n* = 2), tachyarrhythmia (*n* = 2), or suspected myocarditis (*n* = 2). In the remaining 28 patients, CMR was performed in the course of diagnostic workup of CA.

In CMR, LV ejection fraction (EF) differed significantly between unknown-cause OHCA patients and those with a definite cause (49% [IQR: 43–56] vs. 41% [IQR: 33–50], *p* = 0.030), as did EDVi, ESVi, and MM (all *p* < 0.03).

LGE was found in 39 patients (48%, 30 ischemic vs. 9 non-ischemic pattern), with unknown-cause OHCA patients presenting significantly less common with LGE (9% vs. 72%, *p* < 0.001). An ischemic LGE pattern was found in 6% of unknown-cause patients (*n* = 2/32, in both cases small-focal areas) and in 56% of definite-cause patients (*n* = 28/50). Overall, MAD patients showed LGE significantly less often (*n* = 5/24, 21% vs. *n* = 34/58, 59%, *p* = 0.002) compared to patients without MAD presence. No MAD patient showed papillary muscle enhancement. A detailed list of CMR measurements is shown in Table 1 and 2, respectively.

### Rhythmological features

A detailed list of rhythmological features is shown in supplementary Table 2. At the index event, the initial rhythm (recorded in 81 patients, 94%) showed no difference between OHCA patients with unknown and definable cause (*p* = 0.155) or between patients with and without MAD (*p* = 0.051), with MAD patients presenting exclusively with ventricular fibrillation (VF). Post-CPR-ECG on the day of index CA was available in 79 patients (92%) and differed significantly between unknown-cause OHCA patients and those with definable cause concerning repolarization disorders (*p* = 0.020), primarily concerning ST elevation (12/47, 26% vs. 1/32, 3%), with the other entities encompassing unspecific repolarization disorders. There was no significant difference regarding rhythm (*p* = 0.568), electrical heart axis (*p* = 0.349), P-wave morphology (0.211), bundle branch blocks (*p* = 0.337), pathological Q-waves (*p* = 0.843), signs of hypertrophy (*p* = 0.387), and specific time intervals (PQ, QRS, QT/QTc, all *p* > 0.2). Data about rhythmologic events before and after index event as well as during hospitalization are shown in the supplements.

### Laboratory analysis

Within laboratory parameters, unknown-cause OHCA patients showed significantly lower values of serum creatinine (difference: 0.25 mg/dl, *p* < 0.001), calcium (0.1 mmol/l, *p* = 0.009), peak troponin T (409 ng/ml, *p* = 0.017), and peak N-terminal pro-brain-type natriuretic peptide (565 ng/l, *p* = 0.048). Lab results are shown in Table 1.

## Discussion

This study is the first to investigate the role of MAD particularly in OHCA patients undergoing CMR imaging. Our results are as follows: when screened and diagnosed via CMR, (a) MAD is common in patients with unknown-cause OHCA, whilst (b) it is far less common in patients with a definable cause of OHCA; (c) MAD patients in our CMR study showed generally less comorbidities for cardiovascular events; however, (d) MAD was revealed to be an independent marker for unknown-cause OHCA after adjustment for age, hypertension, and hypercholesterolemia.

### Prevalence of unknown-cause OHCA

In the present analysis, no definite cause for CA could have been found in 40% of OHCA patients despite profound diagnostics. This number exceeds the observations of a German register study investigating 33,772 OHCA patients between 2007 and 2017. In that study, the proportion of unknown-cause OHCA was 17% [16]. A possible explanation for this discrepancy is the fact that in most cases of definable-cause OHCA (e.g. myocardial infarction), there is no general recommendation for further investigation via CMR [17]. Furthermore, due to its limited availability, CMR is usually only performed when the most common reasons for CA can be excluded beforehand. This additionally increases the percentage of unknown-cause OHCA in our study, in which, however, performance of CMR is a central inclusion criterion.

### MAD in unknown-cause OHCA

In this study, MAD was defined as end-systolic disjunction extent of at least 1 mm, referring to an important forerunner study by Dejgaard et al. [3]. This approach can currently be regarded as quite strict definition of MAD, as many other studies tended to define MAD as disjunction of any extent [7, 11, 18]. However, in some rare studies, also larger cut-offs can be found, such as 2 mm [19] and 5 mm [20]. As the minimum MAD in this present study was 2.8 mm, shifting the threshold to 2 mm would have had no effect on the outcome; however, a threshold of 5 mm would have decreased the MAD prevalence to 17% (*n* = 15, 12 with unknown-cause OHCA, 35% vs. 3 with definable-cause OHCA, 6%, *p* < 0.001). Moreover, a MAD cut-off of 8.5 mm (which was shown to predispose for the development of non-sustained ventricular tachycardia [21]) still yielded a significant result (MAD < 8.5 mm, *n* = 7: 86% unknown-cause vs. > 8.5 mm, *n* = 79: 34% unknown-cause, *p* = 0.009).

One main finding of our study was that MAD was diagnosed significantly more often in unknown-cause OHCA patients, while these patients generally showed distinctly less comorbidities, especially in terms of age, BMI, blood pressure, hypercholesterolemia, and CAD prevalence. According to a cohort study by Essayagh et al. in 595 MVP patients, the presence of disjunction was an independent risk factor for the occurrence of arrhythmic events in the long-course [22]. This finding is in line with a study by Dejgaard et al., which found severe arrhythmic events in 12% of MAD patients and postulated MAD to be an arrhythmogenic risk factor itself, independent of concomitant prolapse [3]. What is more, a large CMR-register study by Zugwitz et al. in 2022 numbered a mean MAD-extent of 3.4 mm in healthy subjects with MAD [23], which is clearly lower than in our cohort (7.1 ± 3.6 mm), indicating that there might be an association between extent and clinical relevance. Accordingly, multivariate logistic regression analysis in the present study revealed MAD to be an independent marker of OHCA of unknown cause after adjustment for age, hypertension, and hypercholesterolemia. The latter three all represent classical risk factors of ischemic heart disease, which mirrors the high prevalence of myocardial infarction in the definable group. There are hardly any other data available about the role of MAD in OHCA. However, a study by Lee et al. investigating the association of MVP and severe arrhythmias indicated that systolic curling motion in MAD was a strong and independent predictor of these events [15]. In the present study, systolic curling motion was more common in unknown-cause OHCA patients; however, this difference was not significant, which is probably due to the small number of MAD in definable-cause OHCA patients.

### Features of unknown-cause OHCA

Besides MAD, female sex has proven to be a strong prognostic marker for unknown-cause OHCA (and besides the only other significant marker for unknown-cause OHCA in the univariate analyses), with 74% of these patients being female. Referring to the above-mentioned register study, almost 65% of all OHCA patients were male, which is in line with our study (58%). However, the percentage of women with unknown-cause OHCA in that register study was 40%. This is most likely due to the high rate of cardiac events in the definable-cause group (83%), which is accordingly more common in men [16]. Then, although patients in the unknown-cause group in general had structurally normal hearts, some of them still showed an EF below 40%. A sensible explanation for this phenomenon can be found in a study by Gonzalez et al., describing a marked decrease of LV-EF up to 25% due to CA, hinting that perhaps these patients with lower EF at CMR had a normal ventricular function pre-CPR [24]. Additionally, it can be assumed that the partly quite short interval between CPR and CMR also plays a non-neglectable role here, as the LV function underlies a high variability during the first few days after cardiac recovery, which was shown by Kalra et al. in OHCA patients via echocardiography [25]. Another finding, which is probably a result of the high frequency of cardiac triggers for CA in the present study, is that LGE was significantly less common in unknown-cause OHCA and in MAD patients. LGE was found to be a strong predictor for definable-cause OHCA. The percentage of patients with LGE in the definable-cause group (72%) is in line with a study by Neilan et al., detecting LGE in 71% of a patient cohort of 137 CA survivors [26]. Contrary, the proportion of patients showing LGE in the unknown-cause group was less than a tenth. This could be due to the young age of patients in this group as well as the low risk profile.

### Epidemiologic features of MAD

In the present study, MAD was evident in 28% of patients in at least one segment of the posterior mitral leaflet. This is approximately in line with three studies reporting the prevalence of MAD via transthoracic echocardiography in MVP patients (MAD in 22%) [27], via 3D-TEE in a mixed-patients cohort (27%) [15] and via CMR in myxomatous mitral valve disease (35%) [7]. However, according to a recently published study by Toh et al., investigating the prevalence of MAD in a population of 98 patients without structural heart disease via CT, the true prevalence of MAD could be up to 96% [6]. This marked difference to our present study could be at least partly due to the underlying examination method, as CT shows a higher spatial resolution than CMR, which also manifests itself in a larger median MAD-extent in our study (5.7 mm vs. 3.0 mm in Toh et al.).

The ratio of 75% women in MAD patients is in line with a study by Perazzolo Marra et al., describing MAD as a constant feature of arrhythmogenic MVP [4]. According to a large investigation of MVP prevalence in the course of the Framingham Study, MVP in general was shown to be a feature mainly affecting young women [28]. As MAD is very often still accompanied by MVP, this would be in agreement with our data. However, data about sex distribution in MAD are currently rather inconsistent [2] and studies still lacking. Another common feature of MVP is thickening of the mitral leaflets [4], which was shown to be best described via CT or echocardiography [29]; in our cohort, mitral leaflet thickening ≥ 5 mm (i.e. suggesting Barlow’s disease [30]) was not detected (long-axis cine images, end-diastole), with a median thickness of 1 mm and a maximum of 3 mm.

### MAD and arrhythmias

Interestingly, the difference in initial ECG findings at CPR between patients with and without MAD was of borderline significance, with all 24 MAD patients showing VF at first medical contact. Overall, 84% of patients in the present study initially presented with a shockable rhythm, which is in line with a study by Majewski et al. investigating 871 OHCA patients that survived the first 30 days after CPR [31]. In the first post-CPR-ECG, the main difference between definable-cause and unknown-cause lies in the presence of repolarization disorders, being primarily due to the high ratio of myocardial infarctions showing ST elevation in the first group, with the remaining entities being rather unspecific repolarization abnormalities. There are no data available about arrhythmias requiring CPR in MAD; however, as this study’s MAD patients were distinctly younger than patients without MAD and showed less risk factors (i.e. diabetes, hypertension, hypercholesterolemia, CAD), these findings hint that the disjunction itself bears arrhythmogenic potential, especially in favour of ventricular arrhythmias [3]. Further, although being more and more regarded as arrhythmogenic entity itself, it is not yet clear why the presence of MAD seemingly predisposes for the development of severe arrhythmic events. Some studies postulate fibrosis of the myocardial wall or the papillary muscles as a reaction to myocardial stretch generated by a hypermobile mitral valve apparatus and a contingently prolapsing leaflet as the primary pathophysiology of MAD arrhythmic syndrome [32, 33], which can pithily be summarized as ‘hypermobility-stretch-fibrosis-sequence’. Another hypothesis involving damage or tissue change of the cardiac conduction system has not yet been sufficiently investigated [3, 34], but would explain the increased risk of arrhythmic events in MAD patients even without the presence of MVP [3] or, as shown in our study, LGE. The fact that only 9% of unknown-cause patients in our study showed replacement fibrosis is probably due to a compound of these and maybe still unknown pathophysiological mechanisms that result in arrhythmias even before being measurable. Then, if the currently more prevalent idea of a sequence consisting of a hypermobile mitral valve apparatus causing persistent myocardial stretch, which itself leads to myocardial fibrosis, proves right or at least partially truthful, future considerations regarding treatment could involve interventional or surgical remedying of the primarily underlying hypermobility before the development of clinically relevant fibrosis [35].

### Study limitations

We acknowledge that this study bears some limitations, with the most important being its retrospective nature, which results in partly incomplete patient history records and further course after discharge. For example, genetic testing or endomyocardial biopsy was only performed in eight of the unknown-cause OHCA patients, as in clinical routine most genetic/congenital causes for CA can often be excluded either via ECG (long/short QT syndrome, Brugada syndrome, early repolarization syndrome) or via CMR (primarily arrhythmogenic right ventricular cardiomyopathy) [36]; nevertheless, a wider use of genetic testing would have raised the validity of our study. Furthermore, the selected patient population is subject to a certain selection bias, as the percentage of patients with unknown-cause OHCA was overproportionally high, due to the availability of CMR imaging being a central inclusion criterion and the less common referral to CMR in definite-cause OHCA. Another issue associated with our retrospective approach was that not all patients had CMR after OHCA—however, when excluding those with CMR prior to CA, MAD would still be more common in unknown-cause OHCA (57% vs. 15%, *p* < 0.001). Then, a probably very helpful tool and valuable addition to our analysis concerning tissue characterization in MAD could be parametric myocardial mapping. However, these sequences were not obtained in many patients due to these patients having been scanned before mapping sequences were commercially available. Lastly, CMR protocols were not entirely uniform due to the fact that CMR was primarily performed as a part of clinical routine rather than a scientific study; however, all patients were adequately evaluable in terms of MAD and cardiac function.

## Conclusion

MAD is a common feature in OHCA patients without a definable substrate for CA. MAD patients were younger, more often female, and typically presented with a lower risk profile. However, the mere presence of MAD seems to be an independent factor of OHCA without clear trigger. Further research to characterize and understand the role of MAD in CA is needed.

### Supplementary Information

Below is the link to the electronic supplementary material.

Supplementary file1 (DOCX 587 KB)

## Data Availability

The data underlying this article will be shared on reasonable request to the corresponding author.

## Abbreviations

BMI: Body mass index
CA: Cardiac arrest
CAD: Coronary artery disease
CI: Confidence interval
CMR: Cardiovascular magnetic resonance imaging
CPR: Cardiopulmonary resuscitation
CT: Computed tomography
CTA: Computed tomography angiography
ECG: Electrocardiogram
EDV: Enddiastolic volume
EDVi: Enddiastolic volume indexed by body surface area
ESV: Endsystolic volume
ESVi: Endsystolic volume indexed by body surface area
IQR: Interquartile range
LGE: Late gadolinium enhancement
LV: Left ventricle/ventricular
MAD: Mitral annular disjunction
MM: Myocardial mass
MVP: Mitral valve prolapse
OHCA: Out-of-hospital cardiac arrest
OR: Odds ratio
VF: Ventricular fibrillation
