# Supplementary material for: Mitral annular disjunction in out-of-hospital cardiac arrest patients—a retrospective cardiac MRI study
Source: Clin Res Cardiol. 2024 Apr 11;113(5):770–80. doi: 10.1007/s00392-024-02440-3 (PMC11026248; doi:10.1007/s00392-024-02440-3)
Supplement: Supplementary file 1 — Supplementary file1 (DOCX 587 KB) [file 392_2024_2440_MOESM1_ESM.docx]

Supplemental Material

**Cardiovascular Magnetic Resonance Imaging Protocol.**

The standard CMR-protocol included high-resolution cine-images in long- and short axis view covering the left ventricle (LV), using a balanced steady state free precession sequence with retrospective ECG-gating (slice thickness: 8mm, interslice gap: 2mm, echo time: 1.19ms, repetition time: 2.83ms, 22 lines per segments, median temporal resolution: 39.9 ms (interquartile range (IQR): 38.4-46.1), frame rate: 25 frames per second, flip angle: 70°, field of view: 380x310mm, matrix: 320x260, voxel size: 2.6x1.8x8.0mm³, parallel imaging mode: GRAPPA (generalized autocalibrating partial parallel acquisition) with acceleration factor 2). ECG-triggered, phase-sensitive inversion recovery sequences were used to obtain late gadolinium enhancement (LGE) images 15-20 minutes after application of a 0.2 mmol/kg body mass gadolinium-contrast bolus. Standard software (Circle Cardiovascular Imaging, Calgary, Canada) was used for post-processing-analyses with semi-automatic detection of LV and right ventricular endo- and epicardial borders. Papillary muscles were excluded from myocardial mass (MM) and included in the LV volume. End-diastolic volume (EDV) and end-systolic volume (ESV) were then divided by the body surface area [m²] to obtain indexed values (EDVi and ESVi). Contrast agent was administered in 82 patients (95%).

Dichotomized at a median LV-EF of 49%, patients with a lower LV-EF had their scan 4.8 days after CPR, while the patient group with an LV-EF above the median had their scan 7.4 days after CPR (p=0.013). Furthermore, LV-EF correlated significantly with days between CPR and CMR (spearman’s rho: 0.574, p<0.001).

**Rhythmological history and further course.**

According to patient files, a total of 7 patients (8%) showed a history of arrhythmological conditions prior to the index event. During hospitalization, arrhythmic episodes were recorded in 14 patients (16%). In the aftermath of index hospitalization, over a median observation period of 3.3 years (IQR: 1.8-6.7), new episodes of arrhythmia were documented in 8 patients (9%). Between unknown-cause and definable-cause OHCA-patients, no significant difference concerning peri-CPR rhythmological events was shown (before: p=0.494, during hospitalization: p=0.646, afterwards: p=0.444). The same accounts between patients with and without MAD (before: p=0.733, during hospitalization: p=0.912, afterwards: p=0.304).

**Supplemental Table 1.** Uni- and multivariable analyses

|  | **OR (95% CI)** | **p-value** |
| --- | --- | --- |
| **Univariable Analysis** |  |  |
| MAD presence | 11.91 (3.79-37.37) | **<0.001** |
| MAD-extent, mm | 1.41 (1.17-1.71) | **<0.001** |
| MAD - ≥2 segments affected | 21.25 (2.57-175.64) | **0.005** |
| Age at CPR, yrs | 0.96 (0.93-0.98) | **0.002** |
| Female Sex | 10.35 (3.77-28.47) | **<0.001** |
| BMI, kg/m² | 0.88 (0.79-0.98) | **0.021** |
| Diabetes | 0.30 (0.06-1.49) | 0.142 |
| Hypertension | 0.22 (0.09-0.59) | **0.002** |
| Hypercholesterolemia | 0.20 (0.07-0.60) | **0.004** |
| Atrial Fibrillation | 0 (0) | 0.999 |
| CAD (CT or invasively) | 0.11 (0.04-0.30) | **<0.001** |
| Creatinine, mg/dl | 0.08 (0.01-0.50) | **0.007** |
| Calcium, mmol/l | 0.01 (0.00-0.32) | **0.009** |
| Peak hs-Troponin T, ng/ml | 1.00 (0.99-1.00) | 0.051 |
| Peak NT-proBNP, ng/l | 1.00 (1.00-1.00) | 0.766 |
| EF, % | 1.04 (0.99-1.09) | 0.091 |
| EDVi, ml | 0.98 (0.96-0.99) | **0.044** |
| ESVi, ml | 0.98 (0.96-0.99) | **0.037** |
| Myocardial Mass, g | 0.97 (0.95-0.99) | **0.001** |
| LGE | 0.04 (0.01-0.15) | **<0.001** |
| **Multivariable Analysis** |  |  |
| *Model 1* | **n = 84** |  |
| - MAD presence | 8.49 (2.37-30.41) | **0.001** |
| - Age at CPR, yrs | 1.00 (0.97-1.04) | 0.842 |
| - Hypertension | 0.42 (0.12-1.43) | 0.164 |
| - Hypercholesterolemia | 0.36 (0.10-1.27) | 0.111 |
| *Model 2* | **n = 84** |  |
| - MAD presence | 6.26 (1.72-22.76) | **0.005** |
| - Female Sex | 4.61 (1.41-15.09) | **0.011** |
| - Hypertension | 0.54 (0.17-1.75) | 0.306 |
| - Hypercholesterolemia | 0.61 (0.16-2.23) | 0.451 |
| *Model 3* | **n = 81** |  |
| - MAD presence | 5.03 (1.29-19.71) | **0.020** |
| - Female Sex | 3.68 (1.09-12.39) | **0.036** |
| - CAD (CT or invasively) | 0.30 (0.09-1.05) | 0.059 |
| - Hypertension | 0.70 (0.20-2.45) | 0.576 |
| *Model 4* | **n = 83** |  |
| - MAD presence | 5.23 (1.33-20.53) | **0.018** |
| - Age at CPR, yrs | 1.00 (0.96-1.05) | 0.869 |
| - Female Sex | 4.42 (1.27-15.37) | **0.020** |
| - CAD (CTA or invasively) | 0.24 (0.06-0.93) | **0.039** |
| *Model 5* | **n = 79** |  |
| - MAD presence | 9.05 (1.57-52.12) | **0.014** |
| - Female Sex | 2.58 (0.63-10.61) | 0.189 |
| - CAD (CT or invasively) | 0.53 (0.12-2.34) | 0.403 |
| - LGE | 0.04 (0.01-0.25) | **<0.001** |

*BMI: body mass index, CAD: coronary artery disease, CPR: cardio-pulmonary resuscitation, CT: computed tomography, EDVi: indexed end-diastolic volume, EF: ejection fraction, ESVi: indexed end-systolic volume, hs: high-sensitive, LGE: late gadolinium enhancement, MAD: mitral annular disjunction, NT-proBNP: N-terminal pro-B-type natriuretic peptide*.

**Supplemental Table 2.** Rhythmological features.

|  | **All patients (n=86)** | **definable-cause (n=52)** | **unknown-cause**  **(n=34)** | **p-value** |
| --- | --- | --- | --- | --- |
| **Initial Rhythm at CPR (81/86 patients), n(%)**   - VF - VT - Asystole - PEA | 67 (78)  5 (6)  5 (6)  4 (5) | 37 (71)  3 (6)  3 (6)  4 (8) | 30 (88)  2 (6)  2 (6)  0 (0) | 0.155 |
| **Repolarisation at baseline ECG (79/86 patients), n(%)**   - ST-elevation - ST-depression - terminal negative T - preterm. negative T | 13 (16)  4 (5)  11 (14)  9 (11) | 12 (26)  1 (2)  4 (9)  7 (15) | 1 (3)  3 (9)  7 (22)  2 (6) | **0.020** |
| **Events in patient history, n(%)**   - VT - Frequent ES - BBB | 2 (2)  3 (3)  2 (2) | 2 (4)  1 (2)  1 (2) | 0 (0)  2 (6)  1 (3) | 0.507 |
| **Events during hospitalisation, n(%)**   - VF - VT - Asystole - PEA - AF | 5 (6)  6 (7)  1 (1)  0 (0)  2 (2) | 4 (8)  3 (6)  1 (2)  0 (0)  1 (2) | 1 (3)  3 (9)  0 (0)  0 (0)  1 (3) | 0.646 |
| **Events after discharge, n(%)**   - VF - VT - AF | 5 (6)  2 (2)  1 (1) | 2 (4)  1 (2)  0 (0) | 3 (9)  1 (3)  1 (3) | 0.444 |

*AF: atrial fibrillation, BBB: bundle branch block, CPR: cardio-pulmonary resuscitation, ES: extrasystole, PEA: pulseless electrical activity, VF: ventricular fibrillation, VT: ventricular tachycardia.*

**
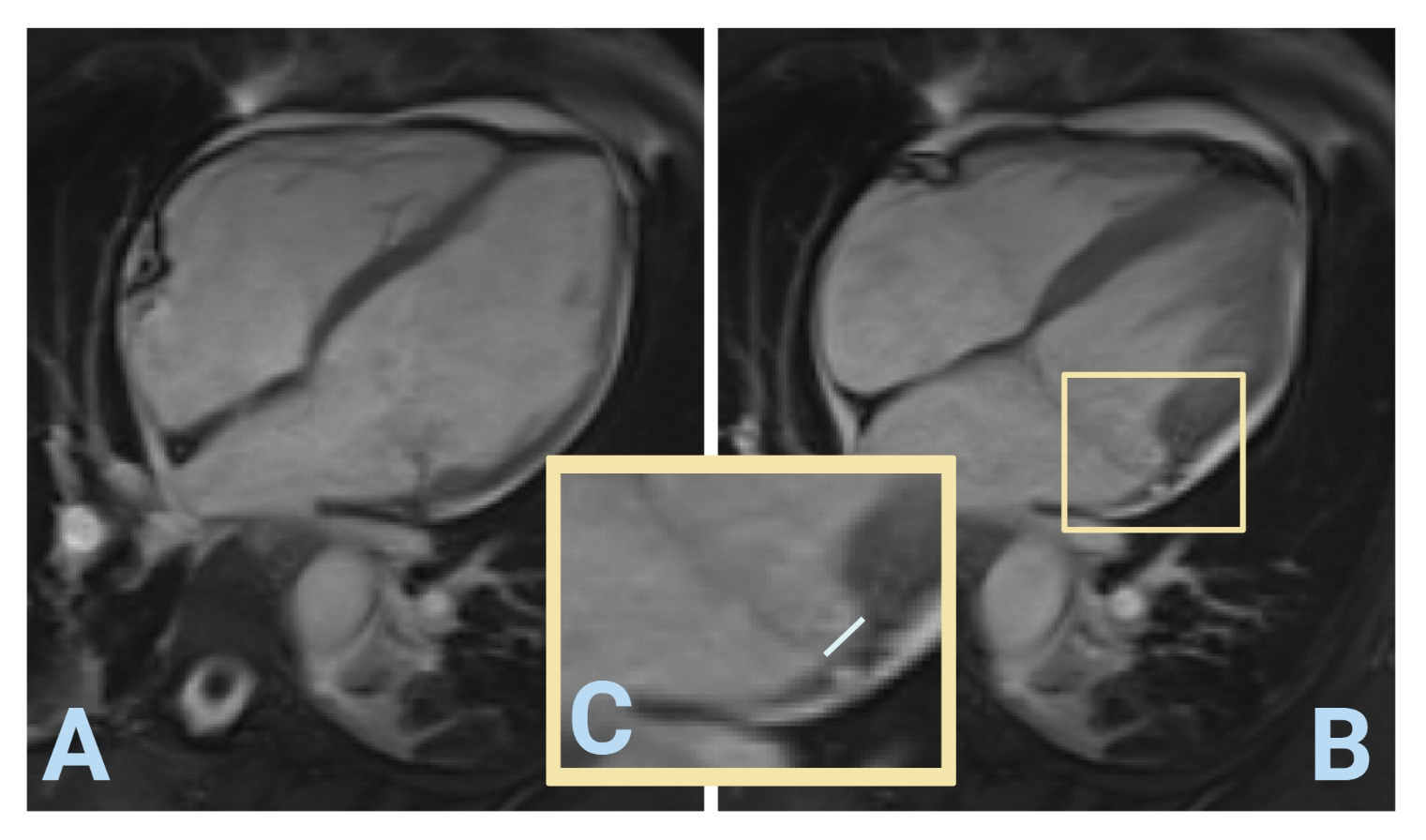
Supplemental Figure 1.** The detection of mitral annular disjunction and the measurement of its extent depends very much on the correct phase: this patient showed a marked disjunction of the P1-segment in end-systole (B), which is not visible at end-diastole (A). Panel (C) shows the positioning of the measuring tool as was used in this current study.
